# Supplementary material for: Sequencing Therapy for Optimal Response in Mirikizumab (STORM)-study: A tertiary referral center study on patients with therapy-refractory ulcerative colitis
Source: PLoS One. 2025 Oct 24;20(10):e0334897. doi: 10.1371/journal.pone.0334897 (PMC12551913; doi:10.1371/journal.pone.0334897)
Supplement: S1 Table — with percentages for categorical variables, mean ± standard deviation for normally distributed data, and median with interquartile range for non-normally distributed data. (PDF) [file pone.0334897.s001.pdf]

S1 Table. Characteristics of the anti-TNF-treated and anti-TNF-naïve patients at baseline with percentages for categorical variables, mean ± standard deviation for normally distributed data, and median with interquartile range for non-normally distributed data

	**Anti-TNF pretreatment**		**p value**
	**Yes**	**No**	
Age<br>n, mean (SD)	18, 38.00 (12.18)	12, 52.17 (13.12)	0.125***
Female sex<br>n (%)	13 (48.15)	9 (36.0)	0.376**
BMI<br>n, mean (SD)	18, 23.36 (3.86)	12, 27.82 (6.70)	0.306***
Disease duration (years)<br>n, median (IQR)	18, 8.5 (11)	12, 6.5 (14)	0.524*
SCCAI<br>n, median (IQR)	18, 5.5 (5)	12, 5.0 (8)	0.460***
FC levels<br>n, median (IQR)	18, 556.0 (1961)	12, 1061 (1716)	0.243*
CRP levels<br>n, median (IQR)	18, 0.43 (1.4)	12, 0.26 (1.63)	0.591*
Weight<br>n, median (IQR)	18, 71.5 (25.3)	12, 81 (43.6)	0.213*

BMI, body mass index; CRP, C-reactive protein; FC, fecal calprotectin; *IQR,* interquartile range; SCCAI, Simple Clinical Colitis Activity Index; SD, standard deviation. *Wilcoxon–Mann–Whitney U test, **chi-square test, ***t-test
