# Supplementary material for: Sequencing Therapy for Optimal Response in Mirikizumab (STORM)-study: A tertiary referral center study on patients with therapy-refractory ulcerative colitis
Source: PLoS One. 2025 Oct 24;20(10):e0334897. doi: 10.1371/journal.pone.0334897 (PMC12551913; doi:10.1371/journal.pone.0334897)
Supplement: S2 Table — with percentages for categorical variables, mean ± standard deviation for normally distributed data, and median with interquartile range for non-normally distributed data. (PDF) [file pone.0334897.s002.pdf]

S2 Table. Characteristics of the JAK inhibitor-treated and JAK inhibitor-naïve patients at baseline with percentages for categorical variables, mean ± standard deviation for normally distributed data, and median with interquartile range for non-normally distributed data

	**JAK pretreatment**		**p value**
	**Yes**	**No**	
Age<br>n, mean (SD)	10, 38.70 (13.09)	20, 46.15 (14.41)	0.291***
Female sex<br>n (%)	9 (52.9)	13 (37.1)	0.279**
BMI<br>n, mean (SD)	10, 24.63 (4.44)	20, 25.40 (6.11)	0.563***
Disease duration (years)<br>n, median (IQR)	10, 8.5 (11)	20, 7.0 (14)	0.839*
SCCAI<br>n, median (IQR)	10, 5.5 (5)	20, 5.0 (6)	0.877***
FC levels<br>n, median (IQR)	10, 1590.5 (1696)	20, 480 (1922)	0.495*
CRP levels<br>n, median (IQR)	10, 0.29 (1.27)	20, 0.47 (1.72)	0.353*
Weight<br>n, median (IQR)	10, 78.5 (19.8)	20, 72.5 (24.3)	0.927*

BMI, body mass index*; CRP,* C-reactive protein; FC*,* fecal calprotectin; IQR*,* interquartile range; SCCAI, Simple Clinical Colitis Activity Index; SD, standard deviation. *Wilcoxon–Mann–Whitney U test, **chi-square test, ***t-test
