# Supplementary material for: Sequencing Therapy for Optimal Response in Mirikizumab (STORM)-study: A tertiary referral center study on patients with therapy-refractory ulcerative colitis
Source: PLoS One. 2025 Oct 24;20(10):e0334897. doi: 10.1371/journal.pone.0334897 (PMC12551913; doi:10.1371/journal.pone.0334897)
Supplement: S3 Table — with percentages for categorical variables, mean and standard deviation for normally distributed data, and median with interquartile range for non-normally distributed data. (PDF) [file pone.0334897.s003.pdf]

S3 Table. Characteristics of the ustekinumab-treated and ustekinumab-naïve patients at baseline with percentages for categorical variables, mean and standard deviation for normally distributed data, and median with interquartile range for non-normally distributed data

	**Ustekinumab pretreatment**		**p value**
	**Yes**	**No**	
Age<br>n, mean (SD)	10, 41.70 (10.74)	20, 44.65 (15.83)	0.761***
Female sex<br>n (%)	11 (68,75)	11 (30.56)	0.010**
BMI<br>n, mean (SD)	10, 23.94 (4.41)	20, 25.74 (6.04)	0.801***
Disease duration (years)<br>n, median (IQR)	10, 8.0 (15)	20, 7.5 (12)	0.394*
SCCAI<br>n, median (IQR)	10, 5.0 (5)	20, 5.5 (7)	0.907***
FC levels<br>n, median (IQR)	10, 642.5 (2031)	20, 782.0 (1768)	0.301*
CRP levels<br>n, median (IQR)	10, 0.3 (0.62)	20, 0.58 (1.9)	0.545*
Weight<br>n, mean (SD)	10, 70.10 (13.08)	20, 78.64 (19.74)	0.298***

BMI, body mass index; CRP, C-reactive protein; FC, fecal calprotectin; IQR, SCCAI, Simple Clinical Colitis Activity Index; interquartile range; SD, standard deviation. *Wilcoxon–Mann–Whitney U test, **chi-square test, ***t-test
