# Supplementary material for: Sequencing Therapy for Optimal Response in Mirikizumab (STORM)-study: A tertiary referral center study on patients with therapy-refractory ulcerative colitis
Source: PLoS One. 2025 Oct 24;20(10):e0334897. doi: 10.1371/journal.pone.0334897 (PMC12551913; doi:10.1371/journal.pone.0334897)
Supplement: S4 Table — with percentages for categorical variables, mean ± standard deviation for normally distributed data, and median with interquartile range for non-normally distributed data. (PDF) [file pone.0334897.s004.pdf]

S4 Table. Characteristics of the vedolizumab-treated and vedolizumab-naïve patients at baseline with percentages for categorical variables, mean ± standard deviation for normally distributed data, and median with interquartile range for non-normally distributed data

	**Vedolizumab pretreatment**		**p value**
	**Yes**	**No**	
Age<br>n, mean (SD)	16, 45.38 (14.33)	14, 41.71 (14.36)	0.353***
Female sex<br>n (%)	9 (34.6)	12 (48.0)	0.332**
BMI<br>n, median (IQR)	16, 22.73 (9.92)	14, 24.26 (5.94)	0.803*
Disease duration (years)<br>n, median (IQR)	16, 8.5 (13)	14, 7.0 (13)	0.269*
SCCAI<br>n, median (IQR)	16, 4.5 (6)	14, 6.0 (7)	0.581***
FC levels<br>n, median (IQR)	16, 841.5 (1847)	14, 556.0 (1961)	0.717*
CRP levels<br>n, median (IQR)	16, 0.58 (1.84)	14, 0.33 (1.17)	0.734*
Weight<br>n, median (IQR)	16, 69.5 (39.0)	14, 79.5 (25.3)	0.610*

BMI, body mass index; CRP, C-reactive protein; FC, fecal calprotectin; IQR, interquartile range; SCCAI, Simple Clinical Colitis Activity Index; SD, standard deviation. *Wilcoxon–Mann–Whitney U test, **chi-square test, ***t-test
