# Supplementary material for: Sequencing Therapy for Optimal Response in Mirikizumab (STORM)-study: A tertiary referral center study on patients with therapy-refractory ulcerative colitis
Source: PLoS One. 2025 Oct 24;20(10):e0334897. doi: 10.1371/journal.pone.0334897 (PMC12551913; doi:10.1371/journal.pone.0334897)
Supplement: S5 Table — (PDF) [file pone.0334897.s005.pdf]

S5 Table. Results of the multiple logistic regression analysis of colonic infestation pattern associated with clinical remission

	**Analysis**	
	**p value**	**OR (95% CI)**
Proctitis	0.765	1.291
Rectosigmoiditis	0.619	0.626
Left-sided colitis	0.218	2.619
Pancolitis	0.909	0.931
