# Supplementary material for: Sequencing Therapy for Optimal Response in Mirikizumab (STORM)-study: A tertiary referral center study on patients with therapy-refractory ulcerative colitis
Source: PLoS One. 2025 Oct 24;20(10):e0334897. doi: 10.1371/journal.pone.0334897 (PMC12551913; doi:10.1371/journal.pone.0334897)
Supplement: S6 Table — (PDF) [file pone.0334897.s006.pdf]

S6 Table. Results of the multiple median regression analysis with mixed effects for SCCAI as a dependent variable

Predictors	Analysis	
	**p value**	**Regression coefficient**
Time (week 12)	**<0.001**	**−1.96**
Time (weeks 24–50)	**<0.001**	**−2.96**
Time (weeks 60–80)	**<0.001**	**−2.82**
Anti-TNF pretreatment	0.596	0.311
JAK inhibitor pretreatment	0.679	0.512
Ustekinumab pretreatment	0.575	−0.660
Vedolizumab pretreatment	0.952	−0.029
≥3 biologic agent/small molecule therapies	0.925	0.148

JAK, Janus kinase; SCCAI, Simple Clinical Colitis Activity Index; TNF, Tumor necrosis factor.
