# Supplementary material for: Sequencing Therapy for Optimal Response in Mirikizumab (STORM)-study: A tertiary referral center study on patients with therapy-refractory ulcerative colitis
Source: PLoS One. 2025 Oct 24;20(10):e0334897. doi: 10.1371/journal.pone.0334897 (PMC12551913; doi:10.1371/journal.pone.0334897)
Supplement: S8 Table — (PDF) [file pone.0334897.s008.pdf]

S8 Table. Results of the multiple median regression analysis with mixed effects for FC as a dependent variable

	**Analysis**	
	**p value**	**Regression coefficient**
Time (week 12)	**0.003**	**−587.41**
Time (weeks 24–50)	**<0.001**	**−699.97**
Time (weeks 60–80)	**<0.001**	**−695.15**
Anti-TNF pretreatment	0.142	−238.23
JAK inhibitor pretreatment	0.123	283.07
Ustekinumab pretreatment	0.739	−113.62
Vedolizumab pretreatment	0.0831	32.331
≥3 biologic agent/small molecule therapies	0.549	−217.27

FC, fecal calprotectin JAK, Janus kinase; TNF, Tumor necrosis factor.
