# Supplementary material for: Sequencing Therapy for Optimal Response in Mirikizumab (STORM)-study: A tertiary referral center study on patients with therapy-refractory ulcerative colitis
Source: PLoS One. 2025 Oct 24;20(10):e0334897. doi: 10.1371/journal.pone.0334897 (PMC12551913; doi:10.1371/journal.pone.0334897)
Supplement: Attachment — Submitted filename: Respone to Reviewers.docx [file pone.0334897.s010.docx]

Universitätsmedizin Medizinische Klinik 1 Theodor-Stern-Kai 7 60590 Frankfurt

Vipula Rasanga Bataduwaarachchi

Academic Editor

*PLOS One*

**Direktor: Prof. Dr. S. Zeuzem**

Prof. Dr. I. Blumenstein

Tel.:	+49/(0)69/6301-5441

e-Mail:	blumenstein@em.uni-f	rankfurt.de

Datum:	September 30, 2025

**Direktion**

Sekretariat:	069/6301-4544

Fax:	069-6301-87676

Privatambulanz:	069-6301-6899

Fax:	069-6301-6448

**Zentrale Aufnahme – HOTLINE**

**(ausschließlich für Ärztinnen und Ärzte)**

Tel.:	069/6301-3904 od. 3905

Fax.:	069/6301-4200

**Station 11-1**

Tel.:	069/6301-5012

Fax:	069/6301-6007

**Station B9**

Tel.:	069/6301-5270

Fax:	069/6301-6749

**Station A9**

Tel.:	069/6301-7475

Fax:	069/6301-6748

**Station B3: Intensiv - ICU**

Tel.:	069/6301-7492

Fax:	069/6301-6915

**Spezialambulanzen**

**Gastroenterologie - Hepatologie**

Tel.:	069/6301-5441 od. 7809

Fax.:	069/6301-7412

**Pneumologie - Allergologie**

Tel.:	069/6301-7329

Fax:	069/6301-6392

**Endokrinologie - Diabetologie**

Tel.:	069/6301-5293

Fax:	069/6301-7817

**Ernährungsmedizin**

Tel.:	069/6301-5055

Fax:	069/6301-6288

**Endoskopie**

Tel.:	069/6301-5357

Fax:	069/6301-6247

**Bereichslabor der Med. Klinik I**

Tel.:	069/6301-5893

Fax:	069/6301-7714

Dear Vipula Rasanga Bataduwaarachchi and Reviewer,

we thank you for the constructive and important critiques, which helped to improve our revised manuscript significantly. We have reworked the manuscript. The changes are highlighted yellow and we have addressed your comments point by point below.

**Academic Editor’s remarks**

**1. Please ensure that your manuscript meets PLOS ONE's style requirements, including those for file naming.**

Thank you for aiding us in improving the layout of our manuscript. We have revised the manuscript regarding the style requirements, including those for file naming.

**2. We note that the grant information you provided in the ‘Funding Information’ and ‘Financial Disclosure’ sections do not match.**

**When you resubmit, please ensure that you provide the correct grant numbers for the awards you received for your study in the ‘Funding Information’ section.**

Thank you for making us aware of this discrepancy. We will address this issue.

**3. Thank you for stating the following financial disclosure:**

**“Alica Kubesch was funded by the Bundesministerium  für Forschung, Technologie und Raumfahrt (BMFTR, Federal Ministry of Research, Technology and Space) ‒ 01EO2102 INITIALISE Advanced Clinician Scientist Program.”**

**Please state what role the funders took in the study.  If the funders had no role, please state: "The funders had no role in study design, data collection and analysis, decision to publish, or preparation of the manuscript."**

**If this statement is not correct you must amend it as needed.**

**Please include this amended Role of Funder statement in your cover letter; we will change the online submission form on your behalf.**

Thank you for this important comment. BMFTR (Federal Ministry of Research, Technology and Space) funded the research rotation of Alica Kubesch as a clinician scientist, but did not influence the content of the research. We have amended this in the cover letter.

**4. We note that you received funding from a commercial source: “Abbvie, Amgen, Biogen, Celgene/BMS, Celltrion, Falk Foundation, Fresenius Kabi, Galapagos, Johnson & Johnson, Lilly, Pharmacosmos, Pfizer and Takeda”**

**Please provide an amended Competing Interests Statement that explicitly states this commercial funder, along with any other relevant declarations relating to employment, consultancy, patents, products in development, marketed products, etc.**

**Within this Competing Interests Statement, please confirm that this does not alter your adherence to all PLOS ONE policies on sharing data and materials by including the following statement: "This does not alter our adherence to PLOS ONE policies on sharing data and materials.” (as detailed online in our guide for authors http://journals.plos.org/plosone/s/competing-interests).  If there are restrictions on sharing of data and/or materials, please state these. Please note that we cannot proceed with consideration of your article until this information has been declared.**

**Please include your amended Competing Interests Statement within your cover letter. We will change the online submission form on your behalf.**

We are happy to explain the competing interests in more detail. None of the commercial sources mentioned has any direct influence on the research focus, study design, or even the creation of the manuscript. The lecturer, consulting, or speaker's bureau fees relate to other services that are not related to the current manuscript.

The current study was financed without direct third-party funding. We added this within the initial cover letter and below.

Alica Kubesch reports consulting and lecturer fees from AbbVie, Celgene/BMS, Galapagos, Johnson & Johnson, Takeda, employment and scientitic collaboration from Fraunhofer Institute for Translational Medicine and Pharmacology ITMP and Fraunhofer Cluster of Excellence Immune Mediated Diseases CIMD, research funding by Sanofi and travel grants from Johnson & Johnson, AbbVie, Takeda, Galapagos and Pfizer. Raul Lande reports consulting and lecturer fees from AbbVie, Johnson & Johnson and Takeda and travel grants from Abbvie. Karima Farrag reports consulting and lecturer fees from AbbVie, Amgen, Celgene/BMS, Falk Foundation e.V., Johnson & Johnson, MSD International and Takeda and travel grants from Johnson & Johnson, Pharmacosmos and Lilly. Kathrin Sprinzl reports consulting fees and lecturer fees from AbbVie, Chemomab, Gilead, Ipsen, MSD and project funding from Gilead. ; Florian Alexander Michael reports lecutre fees from Pentax Medical and Dr. Weigert GmBH; Stefan Zeuzem reports consultancy and speaker’s bureau fees from Abbvie, Allergan, Bi-oMarin, Gilead, Intercept, Johnson & Johnson, MSD/Merck, Novo Nordisk, SoBi, and Theratechnologies; Irina Blumenstein reports consulting and lecturer fees from Abbvie, Amgen, Biogen, Celgene/BMS, Celltrion, Falk Foundation, Fresenius Kabi, Galapagos, Johnson & Johnson, Lilly, Pharmacosmos, Pfizer, Takeda, employment and scientitic collaboration from Fraunhofer Institute for Translational Medicine and Pharmacology ITMP and Fraunhofer Cluster of Excellence Immune Mediated Diseases CIMD. and travel grants from Pfizer, Johnson & Johnson, Lilly and Takeda. All other authors have no conflict of interest to disclose.

**5. In the online submission form, you indicated that “The data presented in this study are available on request from the corresponding author. The data are not publicly available due to data protection and ethical regulations.”**

**All PLOS journals now require all data underlying the findings described in their manuscript to be freely available to other researchers, either 1. In a public repository, 2. Within the manuscript itself, or 3. Uploaded as supplementary information.**

**This policy applies to all data except where public deposition would breach compliance with the protocol approved by your research ethics board. If your data cannot be made publicly available for ethical or legal reasons (e.g., public availability would compromise patient privacy), please explain your reasons on resubmission and your exemption request will be escalated for approval.**

We have now provided all results through additional supporting information so that all data is freely available to other researchers.

**6. We note that you have included the phrase “data not shown” in your manuscript. Unfortunately, this does not meet our data sharing requirements. PLOS does not permit references to inaccessible data. We require that authors provide all relevant data within the paper, Supporting Information files, or in an acceptable, public repository. Please add a citation to support this phrase or upload the data that corresponds with these findings to a stable repository (such as Figshare or Dryad) and provide and URLs, DOIs, or accession numbers that may be used to access these data. Or, if the data are not a core part of the research being presented in your study, we ask that you remove the phrase that refers to these data.**

We have removed the phrase “data not shown” and we have added the missing data as supporting information, so that all data is now available to other researchers.

**7. Your ethics statement should only appear in the Methods section of your manuscript. If your ethics statement is written in any section besides the Methods, please delete it from any other section.**

We have now deleted the ethics statement from all sections except the Methods section.

**8. Please include captions for your Supporting Information files at the end of your manuscript, and update any in-text citations to match accordingly. Please see our Supporting Information guidelines for more information:** [**http://journals.plos.org/plosone/s/supporting-information**](http://journals.plos.org/plosone/s/supporting-information)

We have now revised the entire Supporting Information section at the end of the manuscript. Thank you for this information.

**9. If the reviewer comments include a recommendation to cite specific previously published works, please review and evaluate these publications to determine whether they are relevant and should be cited. There is no requirement to cite these works unless the editor has indicated otherwise.** 

There was no corresponding comment from the reviewers.

**Reviewer #1 remarks**

**1. While the retrospective study is acknowledged, the inclusion criterion of “≥3 infusions” requires further clarification.**

This indeed seems confusing; we have have adjusted the statement (line 105).

**2. Clinical remission was indicated by SCCAI ≤4 and biochemical remission by FC ≤250 µg/g. No endoscopic remission data was included. Even in retrospective settings, lack of endoscopic correlation limits clinical value; this should be addressed in the debate.**

Thank you very for this important comment. The issue of the missing endoscopic endpoint has now been emphasized more strongly in the discussion (lines 452-456)

**3. The statistical models (mixed effects logistic regression, median regression) are appropriate, however the paper might benefit from more justification and how patient-level random effects were included.**

We understand the confusion surrounding the application of the statistical model. We have therefore added a further explanation in the Methods section (lines 170-184).

**Reviewer #2 remarks**

**1. In this manuscript, the pronoun "Our" (15 times) was used. In scientific writing, it is better to avoid the pronouns. Please replace them with formal scientific expressions such as "This study," "The present study," or "The current study."**

We agree and have revised the whole manuscript regarding the pronoun “our” and we have replaced it by formal expressions.

**2. Several references [ 5,6,17,18 and 19] are old. Please try to cite more recent references.**

In fact, we have replaced all sources you mentioned with more recent ones, with the exception of source 17. This is the original work, including validation of the SCCAI score.

**3. Kindly I suggest to add conclusion section after the discussion section.**

We absolutely agree. The conclusion section has been added (line 465).

We would like to thank you once again for your constructive criticism and would appreciate your positive feedback.

We look forward to hearing from you at your earliest convenience.

Sincerely,

Prof. Dr. Irina Blumenstein

Goethe University Frankfurt, University Hospital

Medical Clinic 1, Theodor–Stern–Kai 7, 60590 Frankfurt, Germany

Phone No: +49/(0)69/6301-5441

Fax No: +49/(0)69/6301-7412
